# Hidden chromosomal abnormalities in pleuropulmonary blastomas identified by multiplex FISH

**DOI:** 10.1186/1471-2407-6-4

**Published:** 2006-01-05

**Authors:** Benoit Quilichini, Nicolas Andre, Corinne Bouvier, Marie-Anne Chrestian, Angelique Rome, Dominique Intagliata, Carole Coze, Gabriel Lena, Helene Zattara

**Affiliations:** 1 Département de Génétique Médicale – Laboratoire de Cytogénétique Hémato-Oncologique, CHU – Hôpital d'Enfants « La Timone », Bd Jean Moulin, 13385 Marseille Cedex 5, France.; 2 Département d'Oncologie Pédiatrique, CHU – Hôpital d'Enfants « La Timone », Bd Jean Moulin, 13385 Marseille Cedex 5, France.; 3 Département d'Anatomopathologie, CHU – Hôpital d'Adultes « La Timone », Bd Jean Moulin, 13385 Marseille Cedex 5, France.; 4 Département de Chirurgie Pédiatrique, CHU – Hôpital d'Enfants « La Timone », Bd Jean Moulin, 13385 Marseille Cedex 5, France.; 5 FRE-Centre National de la Recherche Scientifique 2737, UFR de Pharmacie, Bd Jean Moulin, 13385 Marseille Cedex 5, France.

## Abstract

**Background:**

Pleuropulmonary blastoma (PPB) is a rare childhood dysontogenetic intrathoracic neoplasm associated with an unfavourable clinical behaviour.

**Cases presentation:**

We report pathological and cytogenetic findings in two cases of PPB at initial diagnosis and recurrence. Both tumors were classified as type III pneumoblastoma and histological findings were similar at diagnosis and relapse. In both cases, conventional cytogenetic techniques revealed complex numerical and structural chromosomal abnormalities. Molecular cytogenetic analysis (interphase/metaphase FISH and multicolor FISH) identified accurately chromosomal aberrations. In one case, *TP53 *gene deletion was detected on metaphase FISH. To date, only few cytogenetic data have been published about PPB.

**Conclusion:**

The PPB genetic profile remains to be established and compared to others embryonal neoplasia. Our cytogenetic data are discussed reviewing cytogenetics PPBs published cases, illustrating the contribution of multicolor FISH in order to identify pathogenetically important recurrent aberrations in PPB.

## Background

Pleuropulmonary pneumoblastoma (PPB) is a rare and aggressive form of childhood's malignancy [1]. PPB exhibits unique histological, biological and clinical characteristics. The term PPB was first proposed by Manivel et al [2]. Histologically, it is characterized by primitive blastemal and mesenchymal elements and lack of malignant epithelial elements. Three histopathological subtypes are described [3]: Type I PPB is exclusively cystic, type II PPB presents intermediate features between purely cystic and solid PPB and type III PPB is a solid tumor without epithelial-lined cystic spaces.

PPB cases can occur within a family environment but PPB has not been described as a well-defined component of a familial childhood cancer/constitutional disease [1,4].

The clinical behavior of this neoplasia remains unpredictable. Despite aggressive multimodal treatment combined with chemotherapy and surgical resection, prognosis of PPB is poor.

We report pathological and cytogenetic findings in two cases of PPB at initial diagnosis and at recurrence. Complex karyotypes were found in case 1 at recurrence and in case 2 at diagnosis. Conventional and molecular cytogenetic techniques were performed and showed complex chromosomal rearrangements.

## Cases presentation

### Clinical data

#### Case 1

A 3-year-old girl had been treated for a type III left PPB in another institution. Initial treatment included surgical removal of the tumor together with 9 courses of chemotherapy in line with to the SIOP MMT95.3B recommendations containing vincristine, adriamycine, carboplatin, etoposide, actinomycin D and ifosfomide. Four years later, she developed, over a week increased vomiting and headaches. A computed tomography of the brain showed a large parietal lobe mass. After performing cerebral and medullar MRI, surgical removal of the tumor was performed. Postoperative computed tomography of the brain showed no residual tumor. Additional exploration for metastasis was negative. Histological diagnosis was PPB type III. She had since received 4 courses of chemotherapy and cerebral radiotherapy was planned to complete treatment. She relapsed 3 months later and was treated with gammaknife radiosurgery. 6 weeks after radiosurgery another cerebral localized relapse occurred. Surgery was performed and another tumour was found on a cerebral MRI 2 months after surgery despite chemotherapy with weekly Navelbine and daily Endoxan. She died shortly after the disease worsened.

#### Case 2

A 4-year old boy was transferred to our department for examination of the left pulmonary mass. Exploratory tests for metastasis were negative. Surgical removal of the tumor was performed. Histological diagnosis was PPB type III. He was treated according to the SIOP MMT95.3B. 29 months after completion of the treatment, routine computed tomography of the lungs showed a left paracardiac mass. Additional tests for metastasis were negative. The mass was surgically removed and histological analysis of the mass lead to the diagnosis of recurrence of type III PPB. The boy is currently being treated with chemotherapy and radiotherapy.

### Pathology

Tumor specimens were put in formalin and paraffin-embedded for histologic and immunohistochemical analysis. Paraffin-embedded tissues were sectioned at 4 μm and stained with hematoxylin-phloxin-safron (HPS). For immunohistochemistry the sections were deparaffined and subjected to antigen retrieval. An automated immunohistochemistry was performed with avidin-biotin-peroxidase complex on a Ventana 320 device (Tucson, AZ, USA) with a Ventana kit (Strasbourg, France) including AEC reagent. The following primary antibodies (dilution and source in parentesis) were used : glial fibrillary acidic protein (GFAP, Dakopatts, 1/2000), S-100 protein (Dakopatts, 1/400), alpha-smooth actin (Dakopatts, prediluted), desmin (Immunotech, prediluted), myogenin (Microm, 1/5).

### Conventional and molecular cytogenetic analysis

Chromosome analysis was performed on tumor fragments obtained from the primary (case2) or recurrent tumors (case1 and 2). For the culture, fragments of sterile tumor tissue were dissociated enzymatically using an RPMI medium containing 0.02% collagenase type II and incubated at 37°C for approximately 1 hour. A cell suspension obtained after centrifugation was sown over 25 cm2 tissue culture Falcon flasks with RPMI medium supplemented with 10% fetal bovine serum and antibiotics. The cells were then incubated at 37°C in a 5% CO2 atmosphere. After the culture entered exponential growth (10 days), chromosomal analysis was carried out using a routine cytogenetic technique. The R-banding technique was used for chromosomal identification. The description of the karyotypes and criteria for clonality follow the recommendations of the International System of Human Cytogenetic Nomenclature (ISCN 1995) [5].

Multicolor fluorescence in situ hybridization (M-FISH) was performed on chromosome preparations from tumor cells. The M-FISH technique was carried out as described by the manufacturer's protocols (Metasystems, Altlussheim, Germany).

For verification and further delineation of the M-FISH findings, Locus-Specific (LSI) and Enumeration (CEP) FISH probes (Vysis, Downers-Grove, IL, USA) were applied to characterize chromosomal abnormalities according the manufacturer's protocols. *TP-53, CEP17, CEP8, C-MYC *and *MYC-N *probes were used.

## Results

### Pathological findings

**Figure 1:**
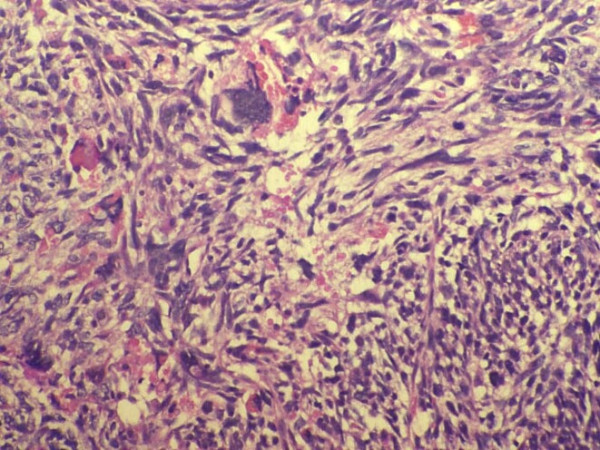
Case n°1: Histology (HES × 100). At recurrence: poorly differentiated blastematous cells with pleiomorphic component.

**Figure 2:**
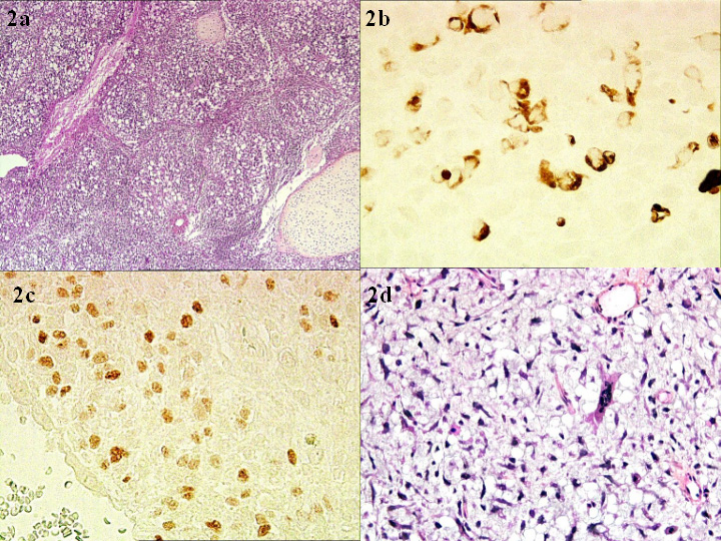
Case n°2: Histology (HES × 20). At diagnosis: poorly differentiated blastematous cells with cartilage (2a). Cytoplasmic desmin (2b) and nuclear myogenin (2c) are observed within the tumor cells. Histology (HES × 100): At recurrence only blastematous areas are observed (2d).

On microscopic examination, both tumors were found to be made of solid sheets of immature cells (figures 1, 2a), some of which were being multinucleated (figure 2d). Mitoses were numerous and necrosis was also present. No cystic area was noted. In case n°1, immunohistochemistry was negative for GFAP and S100 protein. That ruled out a malignant glioma. Myogenic markers were also negative. In case n°2, at the time of the first surgery, a malignant mesenchymal component showing multidirectional differenciation was found to be associated with the blastema. Areas containing immature cartilage and rhabdomyoblastic cells were present. The myogenic differenciation was confirmed by immunohistochemistry. Some cells were positive for desmin and myogenin (figure 2b, 2c). At recurrence case n°2 only showed areas of poorly differenciated blastematous cells (figure 2d). Pathological diagnosis in both cases was PPB type III.

### Cytogenetic data

#### Case 1

At diagnosis no metaphases were found after culture and no frozen material was made available for retrospective molecular cytogenetic techniques.

**Figure 3:**
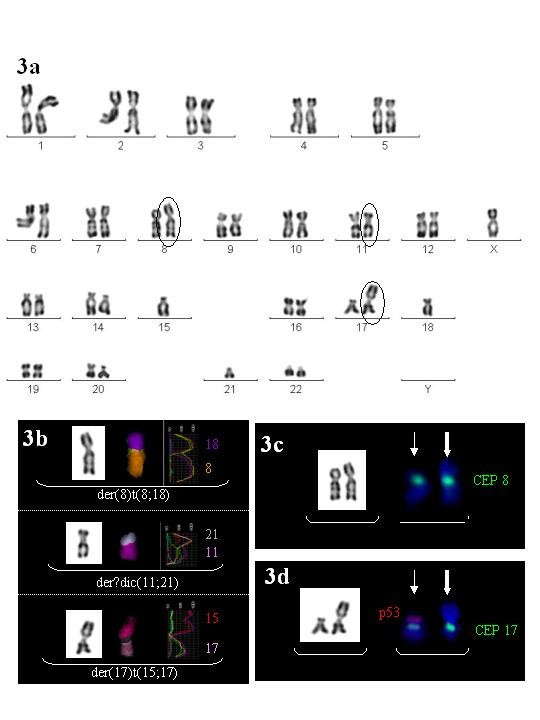
Conventional and molecular cytogenetic data of case 1 at recurrence. 3a: R-banded karyotype: arrows indicate structural clonal abnormalities. 3b: Partial R-banded details associated with partial Multiplex FISH results of each structural chromosomal abnormality. Painting chromosomes by M-FISH are showed in false color view with fluorescence profiles. 3c: Partial metaphase in situ hybridization with a chromosome 8 centromeric probe directly labelled FITC showing one normal chromosome 8 (fine arrow) and one der(8) (thick arrow). 3d: Partial metaphase in situ hybridization with a *TP53 *probe directly labelled Spectrum Orange (SO) and a chromosome 17 centromeric probe directly labelled FITC. This partial metaphase shows one normal chromosome 17 with both FITC and SO fluorescent signals (fine arrow) and one der(17) with an isolated FITC fluorescent signal (thick arrow) indicating a *P53 *gene deletion.

At recurrence, cytogenetic study showed an abnormal and complex karyotype [Figure 3a]. In line with the cytogenetic analysis, we carried out M-FISH [Figure 3b] and metaphase in situ hybridization with CEP 8 probe. We identified an unbalanced translocation between chromosome 8 and 18. The der(8)t(8;18) showed a likely duplication of the 8q11q12 region [Figure 3c]. Metaphase FISH with CEP17 and *TP53 *probes showed a *TP53 *deletion on der(17) identified by M-FISH [Figure 3d]. A dicentric chromosome dic(11;21) with loss of a segment of the short arm of chromosome 11 was revealed. FISH analysis with the *MYCN *and *MYCC *probes did not show showed amplification of both oncogenes (data not shown).

The cytogenetic result was:

38~44,X,-X,der(8)t(8;18)(p1?;q1?),dic(11;21)(p11;p11),-15,der(17)t(15;17)(q1?;p1?3),-18,del(20)(p12pter) [cp11]/81~82<4n>,idemx2 [cp2]/46,XX[7].ish der(8)t(8;18) (D8Z1+,wcp8+,wcp18+),dic(11;21)(wcp11+,wcp21+),der(17)t(15;17)(D17Z1+, wcp15+,wcp17+,TP53-)

#### Case 2

**Figure 4:**
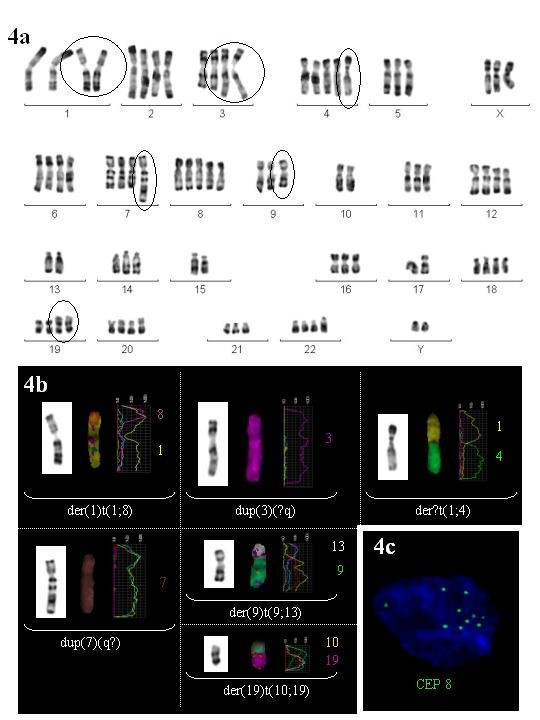
Conventional and molecular cytogenetic data of case 2 at diagnosis and recurrence. 4a: R-banded karyotype from initial tumor characterized by complex numerical and structural clonal abnormalities. Arrows indicate structural clonal aberrations. 4b: Partial R-banded details associated with partial Multiplex FISH results of each structural chromosomal abnormality. Painting chromosomes by M-FISH are showed in false color view with fluorescence profiles. 4c:Interphase in situ hybridization with a chromosome 8 centromeric probe directly labelled FITC from recurrent tumor showing over representation (10 signals) of centromeric region of chromosome 8 : nuc ish 8 cen (D8Z1x10).

At diagnosis, conventional cytogenetic study showed a complex hyperdiploid karyotype (modal number: 91) [Figure 4a]. M-FISH analysis specified complex structural chromosomal abnormalities [Figure 4b].

The cytogenetic result was:

80~109<4n>,XXYY,+X,der(1)t(1;8)(p1?;q1?)x2,dup(3)(?q)x2,+der(?4)t(1;4),-5,dup(7) (q?),+8,-9,der(9)t(9;13),-10,-10,-11,-16,-17,der(19)t(10;19)x2 [cp8]/46,XY[7] .ish der(1)t(1;8)(wcp1+,wcp8+),dup(3)(?q)(wcp3+),der(?4)t(1;4)(wcp1+,wcp4+),dup(7)(q?) (wcp7+),t(9;13)(wcp9+,wcp13+),t(10;19)(wcp10+,wcp19+)

At recurrence karyotype was normal: 46, XY [20]. In order to carry out abnormal clone unidentified by conventional cytogenetic technique, molecular techniques were performed on nuclei by interphase FISH technique using chromosome 8 centromeric region probe. Overrepresentation (8 to 10 signals) of centromeric region of chromosome 8 was confirmed in 32% of interphase nuclei by *in situ *hybridization staining allowing us evoke a related clone (probably a doubled population) from initial tumor [Figure 4c].

## Conclusion

PPB is among the rarest pediatric malignancies. The outcome is particularly unfavourable because of recurrence and/or metastases. The central nervous system represents the main site of metastases [1,6-8].

Only 23 cases with cytogenetic analysis (conventional and/or molecular) have been reported [Additional file 1]. Most of them revealed complex numerical and structural chromosomal abnormalities such as recurrent aneuploidy of chromosome 2 and 8 and structural rearrangement of various chromosomes [9-22]. Interestingly, in 4 cases with conventional cytogenetic studies on a total of 13 cases published, karyotypic description was incomplete (presence of unidentified marker chromosome) [Additional file 1: cases 7, 10, 12 and 18]. Only one case was analyzed by multicolor spectral karyotyping [Additional file 1: case 9].

Both cases we report here represent additional cases studied by conventional and molecular cytogenetic techniques (FISH and M-FISH).

Data found in literature corroborate numerical gains of chromosome 8 as the main recurrent chromosomal abnormality in PPB (22 cases published) [9-13,15-22]. Thus, it may represent a primary event in the genesis of PPB [12]. Few data are available about structural abnormalities of chromosome 8. The two cases we report here showed structural aberration of the either long or short or both arms of chromosome 8 identified by M-FISH. Three other cases showed rearrangement of the long arm of chromosome 8 [Additional file 1 : cases 4, 6 and 18]. Further studies are required to clarify the role of chromosome 8 and to come up with candidate genes in PPB pathogenesis. For instance, PLAG1 which specifically activates transcription is of the highest interest. Indeed, it is involved in the genesis of another embryonal tumour: lipoblastomas, mesenchymal and epithelial neoplasia [23,24].

Amongst pediatric tumors, mutations in *TP53 *gene have been described in sporadic Wilm's tumor, embryonal rhadomyosarcoma and in hepatoblastoma and seemed to be associated with a poor prognosis [25]. However, mutations in the *TP53 *gene are found in a large proportion of adult cancers but are not considered to be specific events of childhood embryonal tumors. In PPB, only one case of *TP53 *deletion was identified by molecular cytogenetic technique [20] but five cases showed a structural rearrangement on 17p [Additional file 1: cases n° 2, 4, 7, 11 and 18]. In these cases, *TP53 *status was not mentioned. Furthermore, Kusafuka et al., using PCR-SSCP method in 2 patients, raised the possibility that p53 inactivation is involved in the pathogenesis and outcome of PPB [26]. In our first case, the *TP53 *gene deletion results in the loss of 17p11pter chromosomal region by an unbalanced translocation t(15;17) [Figure 3d]. The same pattern may be identified in the case of Kelsey et al. with a subsequent loss of 17p material deletion and an unbalanced t(8;17) [12]. This last case was particularly interesting because a clonal evolution was demonstrated from a clone with a trisomy 8 to a clone with a translocation event with a chromosome 17 der(17)t(8;17)(q11.2, p13.1) resulting in a trisomy for the long arm of chromosome 8 and a monosomy for the distal region of chromosome 17 suggesting *TP53 *deletion is a secondary change of importance in the progression of the neoplasia.

Two cases studied by conventional cytogenetic showed a structural rearrangement of the short arm of chromosome 11: a der(11)t(7;11)(q11.2;p11.2) and a dup(11)(p13p15) [Additional file 1: cases 2, 4]. In our first case, we observed a dicentric chromosome dic(11;21) associated with the loss of chromosomal region 11(p11pter). 11p deletion occurred in 38% of cases of nephroblastoma (Wilm's tumor). Two genes seem to be implicated: *WT1 *gene at 11p13 and *WT2 *gene at 11p15 [10,25]. Polymorphism studies showed loss of heterozygosity at 11p13 in 72% of primary embryonal rhabdomyosarcoma [27]. In hepatoblastoma, the region associated with Beckwith-Wiedmann syndrome 11p15.5 could play a role in the development of neoplasia [25]. Although no LOH of markers on 11p13 and 11p15 studies are published to date for PPB, Priest et al. suggest a common genetic pathway among embryonal tumors involving chromosomal region 11p [10]. These authors suggested that PPB should be regarded as a pulmonary dysontogenetic similar to nephroblastoma (Wilm's tumor), neuroblastoma, hepatoblastoma or embryonal rhabdomyosarcoma [10]. Comparative Genomic Hybridization (CGH) findings reported by Roque et al. support the hypothesis that PPB may be tumorigenetically related to embryonal rhabdomyosarcoma [22].

Similarly, Bonner et al. demonstrated that transcriptome wide analysis of gene expression by DNA array technology showed numerous patterns associated with lung development in murines. The authors concluded that the genes identified as relevant to lung development and belonging to three regulatory pathways (Wnt/β-catenin signalling, cell cycle and apoptosis) may be suggested as candidate susceptibility genes for lung tumorigenesis [28]. Chromosome 1p alterations harboring developmental genes related to the Notch and Wnt pathways have been reported also by Garnis et al [29]. Further similar studies in embryonal tumors could be proposed to contribute to our understanding of unusual characteristics in the PPB development.

Chromosomal instability appears to be characteristic of this tumor. Identification by M-FISH technique allowed the identification of recurrent complex structural chromosomal abnormalities. Furthers collegial studies, regrouping PPB cases as in the PPB Registry by Priest and colleagues [30], are required to identify specific chromosomal rearrangements providing a better understanding about the molecular pathogenesis of this aggressive tumor.

## Abbreviations

PPB : Pleuropulmonary blastoma

## Competing interests

The author(s) declare that they have no competing interests.

## Authors' contributions

BQ carried out the cytogenetic and molecular cytogenetic studies and drafted the manuscript.

NA participated in the design and coordination of the study and helped to draft the manuscript.

CB and MAC carried out the histologic and immunohistochemical analysis and helped to draft the manuscript.

AR and CC helped to draft the manuscript.

DI assisted in cytogenetical and molecular techniques and drafted the manuscript.

GL participated in its design and coordination.

HZ conceived the study, participated in its design and coordination and helped to draft the manuscript.

All authors read and approved the final manuscript.

## Pre-publication history

The pre-publication history for this paper can be accessed here:



## Supplementary Material

Additional File 1 Table summarizing cases of pleuro-pneumoblastoma with cytogenetic data. Click here for file
